# Mitochondrial stress as a conceptual interface between bacterial infection and post-infectious metabolic disease

**DOI:** 10.3389/fcimb.2026.1795935

**Published:** 2026-03-06

**Authors:** Nicolás Plaza, Diliana Pérez-Reytor, Gino Corsini, Katherine García, Ítalo M. Urrutia

**Affiliations:** Instituto de Ciencias Biomédicas, Universidad Autónoma de Chile, Santiago, Chile

**Keywords:** bacterial infection, immunometabolism, mitochondrial stress, post-infectious metabolic disease, *Salmonella enterica*, *Vibrio parahaemolyticus*

## Abstract

Mitochondria are central hubs integrating cellular bioenergetics, redox balance, innate immune signaling, and metabolic homeostasis. During bacterial infections, these organelles are recurrent targets of pathogen-derived toxins, secreted effectors, and host inflammatory mediators, leading to a state broadly defined as mitochondrial stress. This stress encompasses alterations in oxidative phosphorylation, mitochondrial dynamics, calcium handling, reactive oxygen species (ROS) production, and activation or disruption of mitochondrial quality control pathways such as mitophagy. In this perspective, we propose mitochondrial stress as a conceptual framework linking bacterial infection and post-infectious metabolic disease. Using enteric bacterial pathogens such as *Salmonella enterica* serovars Typhimurium and Typhi, together with *Vibrio parahaemolyticus*, as conceptual models, we synthesize current evidence showing how distinct bacterial strategies converge on mitochondrial dysfunction and immunometabolic reprogramming of host cells. We argue that, while mitochondrial stress responses may initially support antimicrobial defense, their incomplete resolution may contribute to long-lasting metabolic and inflammatory alterations in epithelial, immune, and metabolic tissues. Persistent mitochondrial dysfunction may contribute to insulin resistance, chronic inflammation, and increased susceptibility to metabolic disease after infection. By framing mitochondrial stress as a central integrator of infection and metabolism, this perspective highlights key knowledge gaps and identifies mitochondria-centered pathways as potential targets to prevent or mitigate post-infectious metabolic sequelae.

## 1 Introduction

Bacterial infections represent acute inflammatory insults that can exert long-lasting effects on host physiology beyond pathogen clearance. Although traditionally viewed as transient events, epidemiological and experimental evidence suggests that bacterial infections may imprint durable metabolic and inflammatory alterations that increase susceptibility to chronic metabolic disease later in life (Medzhitov, 2008; Hotamisligil, 2017; Carey et al., 2018). Epidemiological evidence in humans links severe infectious events to increased metabolic risk. In a large UK Biobank cohort (n = 396,080), hospitalization for infectious diseases was associated with a higher incidence of type 2 diabetes over ~9 years of follow-up, with stronger associations for sepsis and bloodstream infections and a dose-response relationship with infection burden (Zheng et al., 2024). However, the mechanistic by which a temporally limited infection translates into long-term metabolic dysfunction remain poorly defined.

We propose mitochondrial stress as a conceptual framework linking bacterial infection to post-infectious metabolic disease. During infection, mitochondria are repeatedly targeted by pathogen-derived toxins, secreted effector proteins, and host inflammatory mediators, resulting in a coordinated stress response characterized by alterations in oxidative phosphorylation, redox balance, calcium handling, mitochondrial dynamics, and quality control pathways such as mitophagy (Pickles et al., 2018; Tiku et al., 2020). While these mitochondrial stress responses are initially adaptive and support antimicrobial defense, their incomplete resolution following infection may contribute to persistent mitochondrial dysfunction and immunometabolic reprogramming.

Epidemiological studies, largely based on overall infectious burden rather than individual bacterial pathogens, have primarily shown that individuals with pre-existing metabolic disorders are more susceptible to infection (Carey et al., 2018). However, when considered alongside experimental models, these observations raise the possibility that infection-induced inflammation and mitochondrial stress may also contribute to metabolic dysfunction, rather than merely reflecting it (Hotamisligil, 2017; Medzhitov, 2008).

**Figure 1:**
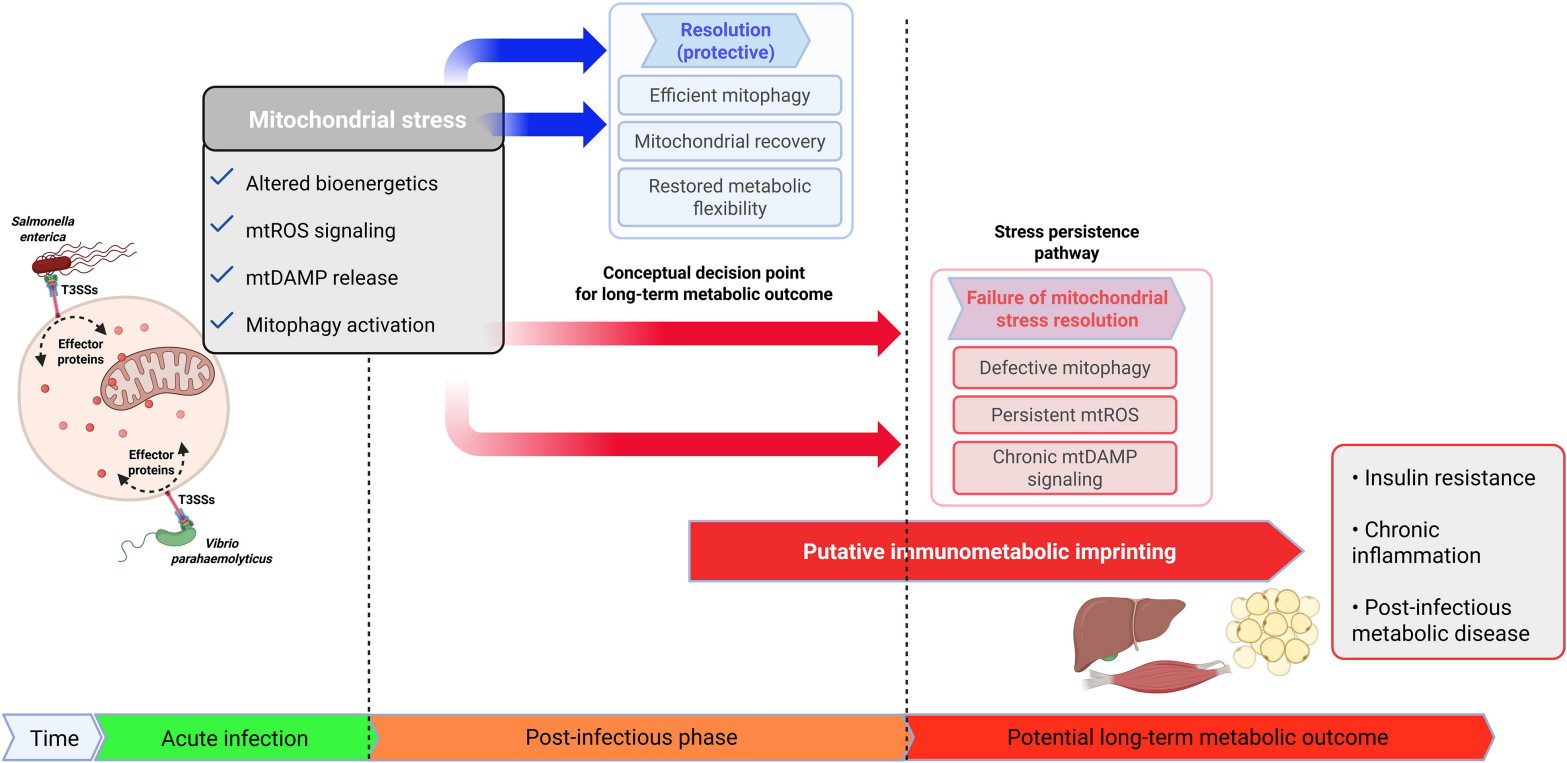
Conceptual framework linking acute enteric bacterial infection, mitochondrial stress resolution and potential long-term metabolic outcomes. This schematic illustrates a hypothesis-driven and conceptual model in which acute enteric bacterial infection induces mitochondrial stress characterized by altered bioenergetics, mtROS signaling, mtDAMP release, and activation of mitochondrial quality control pathways. The figure depicts divergent potential trajectories following infection, including effective mitochondrial stress resolution or incomplete stress resolution associated with persistent mitochondrial dysfunction. The highlighted conceptual decision point represents a putative divergence in mitochondrial stress handling rather than a defined biological switch. Proposed downstream immunometabolic consequences and long-term metabolic vulnerability are shown as possible outcomes, not as established causal relationships. This figure was created using the Biorender application.

Conceptually, this framework proposes that acute infection-induced mitochondrial stress may follow divergent trajectories. Effective mitochondrial quality control can restore bioenergetic homeostasis after pathogen clearance, whereas incomplete repair or dysregulated mitophagy allows stress to persist, leading to bioenergetic inflexibility, chronic low-grade inflammation, and altered immunometabolic signaling. We propose that failure to resolve this mitochondrial stress may represents an important determinant of susceptibility to post-infectious metabolic disease. As summarized in **Figure 1**, bacterial infection-induced mitochondrial stress can be conceptualized as a decision point that may function as a form of immunometabolic memory that shapes metabolic vulnerability.

Mitochondria occupy a central position at the intersection of infection, immunity, and metabolism. As key regulators of oxidative phosphorylation, redox signaling, calcium homeostasis, and biosynthetic metabolism, mitochondria critically shape innate immune responses and systemic metabolic homeostasis (Chandel et al., 2016; Mills et al., 2016, 2017). Consequently, mitochondrial stress emerges as a plausible integrative mechanism through which bacterial infections may exert long-term effects on host metabolic health.

This perspective aims to integrate existing evidence into a hypothesis-driven framework focused on stress resolution versus persistence. Rather than establishing causal relationships, we seek to highlight conceptual connections, unresolved questions, and testable predictions regarding how infection-induced mitochondrial stress may influence metabolic vulnerability. This framework is intended to stimulate future experimental and clinical studies rather than to provide definitive mechanistic conclusions.

## 2 Initiation of mitochondrial stress during bacterial infection

In the initial phase of this conceptual model, bacterial infection may trigger a mitochondrial stress response characterized by coordinated alterations in bioenergetics, redox signaling, and mitochondrial danger signaling. Mitochondria function as central integrators of cellular bioenergetics, redox signaling, and innate immune activation, thereby contributing to metabolic flexibility and tissue homeostasis. Under physiological conditions, mitochondrial oxidative phosphorylation supports efficient ATP production while allowing cells to dynamically adapt substrate utilization in response to nutrient availability, hormonal cues, and energetic demand (Gao et al., 2014; Shen et al., 2022). This metabolic flexibility enables coordinated switching between glucose, fatty acid, and amino acid oxidation and is essential for insulin sensitivity and systemic metabolic health (Petersen et al., 2004; Smith et al., 2018).

During bacterial infection, however, mitochondrial bioenergetics are substantially remodeled. In immune cells, inflammatory activation is accompanied by a shift from oxidative phosphorylation toward aerobic glycolysis, supporting rapid ATP generation and biosynthetic demands associated with antimicrobial responses (O’Neill et al., 2016). While this metabolic reprogramming is adaptive during acute infection, sustained impairment of mitochondrial respiration can compromise metabolic flexibility in non-immune tissues and has been associated with insulin resistance and ectopic lipid accumulation in muscle, liver, and adipose tissue. Mitochondrial dysfunction and impaired switching are further linked to reduced oxidative metabolism and metabolic inflexibility in obesity, insulin resistance, and non-alcoholic fatty liver disease, contributing to systemic metabolic dysfunction (Nunnari and Suomalainen, 2012; Szendroedi et al., 2012; Gao et al., 2014; Feng et al., 2025). Thus, infection-induced mitochondrial dysfunction may represent a plausible initiating context through which transient immune activation could influence long-term metabolic disease risk.

Beyond ATP production, mitochondria function as signaling organelles through the regulated mitochondrial reactive oxygen species (mtROS) generation. At controlled levels, mtROS act as second messengers that amplify innate immune signaling and enhance bactericidal activity downstream of pattern recognition receptor activation (West et al., 2011). mtROS also contribute to activation of inflammatory pathways, including the NLRP3 inflammasome (Zhou et al., 2011). However, excessive or prolonged mtROS production induces oxidative damage to mitochondrial DNA, lipids, and proteins, impairing mitochondrial function and reinforcing a feed-forward loop of inflammation and metabolic stress (Sena and Chandel, 2012).

Oxidative mitochondrial damage facilitates the release of mitochondrial danger-associated molecular patterns (mtDAMPs), including mitochondrial DNA and cardiolipin (Wenceslau et al., 2014). Due to their bacterial ancestry, these molecules activate innate immune sensors such as TLR9 and the cGAS-STING pathway (West et al., 2015). While mtDAMP release can amplifying immune responses during infection, persistent or dysregulated release promotes sterile inflammation and disrupts metabolic homeostasis (Vringer and Tait, 2023). Chronic activation of mtDAMP-sensitive pathways has been implicated in sustained low-grade inflammation, a hallmark of insulin resistance and type 2 diabetes (Hotamisligil, 2017).

Together, alterations in mitochondrial bioenergetics, redox signaling, and danger signaling form an integrated stress response that coordinates host defense but also shapes systemic metabolism. During bacterial infection, these mitochondrial stress responses are tightly regulated to support antimicrobial immunity. However, incomplete resolution of mitochondrial damage may result in persistent bioenergetic inflexibility, exaggerated mtROS signaling, and continued mtDAMP release. This unresolved mitochondrial stress state supports a conceptual framework in which bacterial infection may contribute to long-lasting immunometabolic reprogramming and increased susceptibility to post-infectious metabolic disease.

## 3 Failure of mitochondrial stress resolution and establishment of a maladaptive state

In a subsequent phase of this conceptual model, mitochondrial stress fails to fully resolve following pathogen clearance, giving rise to a maladaptive state marked by persistent bioenergetic impairment, sustained mtROS production, and chronic inflammatory signaling. Furthermore, given its high mitochondrial density and continuous exposure to microbial and dietary cues, the intestinal epithelium represents a site where infection-induced mitochondrial stress may be imprinted and subsequently propagated systemically (Haque et al., 2024). Diverse enterobacteria converge on host mitochondria as major targets during infection, largely independent of their specific virulence strategies or tissue tropism. Members of the Enterobacteriaceae family, including *Salmonella*, *Escherichia coli*, *Shigella*, and *Klebsiella*, as well as related Gram-negative enteric pathogens, deploy toxins, secretion system effectors, and inflammation-inducing mechanisms that perturb mitochondrial bioenergetics, redox balance, calcium handling, and quality control pathways (Haraga et al., 2008; West et al., 2015; Tiku et al., 2020). These mitochondrial perturbations are increasingly recognized not only as contributors to acute epithelial damage and innate immune activation but also as potential contributors to long-lasting metabolic alterations following infection (Hotamisligil, 2017).

Experimental studies using *in vitro* cell culture models show that enterobacterial infections can induce persistent mitochondrial dysfunction in epithelial and immune cells, characterized by impaired oxidative phosphorylation, increased mtROS production, and dysregulated mitophagy (Maurice and Sadikot, 2023; Yang et al., 2025). Importantly, these alterations converge with pathways implicated in insulin resistance, chronic low-grade inflammation, and metabolic disease, as sustained mtROS production and defective mitochondrial quality control promote oxidative and inflammatory stress that interferes with canonical insulin signaling through inhibition of the IRS-PI3K-AKT pathway, while simultaneously activating stress kinases such as JNK and IKKβ and innate immune pathways including NLRP3 inflammasome and NF-κB signaling (Hotamisligil, 2017; Donath et al., 2019). Together, these processes support the view that mitochondrial stress may contribute to immunometabolic dysregulation. Although direct causal links between individual enterobacterial infections and post-infectious metabolic disorders remain incompletely defined, converging evidence supports the view that mitochondria function as long-term immunometabolic “memory hubs” capable of retaining infection-induced stress signals that influence host metabolism well beyond pathogen clearance (Netea et al., 2020).

A central mechanism underlying this convergence is the direct or indirect targeting of mitochondria by bacterial virulence factors. Many Gram-negative pathogens deliver type III secretion system effectors that modulate host signaling pathways converging on mitochondrial dynamics, bioenergetics, and apoptotic control (Haraga et al., 2008; Dean and Kenny, 2009). These effectors rewire host kinase signaling networks, including Akt, MAPK, and NF-κB pathways, which are tightly coupled to mitochondrial metabolism and redox homeostasis (O’Neill et al., 2016). In parallel, bacterial pore-forming toxins and hemolysins disrupt plasma membrane integrity and ion homeostasis, triggering mitochondrial calcium overload, membrane depolarization, and enhanced mtROS production (Gonzalez et al., 2008).

Collectively, these mitochondrial insults activate stress responses that may initially support antimicrobial defense and innate immune activation (West et al., 2011). However, when mitochondrial damage is excessive or incompletely resolved, infected cells become predisposed to sustained bioenergetic impairment, persistent mtROS production, and chronic inflammatory signaling (West et al., 2015; Pickles et al., 2018). This unresolved mitochondrial stress state strengthens the hypothesis that enterobacteria-induced mitochondrial perturbations represent a plausible mechanistic interface between acute infection and post-infectious metabolic disease.

## 4 Pathogen-specific modulation of mitochondrial quality control as a determinant of stress persistence

In a later phase of this conceptual framework, pathogen-specific strategies may intersect with mitochondrial quality control pathways, influencing whether infection-induced mitochondrial stress is efficiently resolved or may become persistently imprinted. *Salmonella enterica* and *Vibrio parahaemolyticus* were selected as illustrative models because they are among the most clinically relevant foodborne bacterial pathogens worldwide and together account for a substantial proportion of the global burden of foodborne disease (Letchumanan et al., 2014; Havelaar et al., 2015), while relying on effector protein repertoires delivered by specialized secretion systems to subvert key host cellular processes during infection. Within the *Salmonella* genus, serovars Typhimurium and Typhi exemplify distinct clinical paradigms: *S*. Typhimurium typically causes an acute, self-limiting gastroenteritis, whereas *S*. Typhi causes typhoid fever, a systemic disease characterized by prolonged infection and, in some cases, chronic carriage (Dougan and Baker, 2014). Despite these divergent outcomes, both serovars are facultative intracellular pathogens that invade epithelial cells and survive within professional phagocytes, thereby engaging host mitochondrial and metabolic pathways from within infected cells (Monack et al., 2004; Haraga et al., 2008).

During infection of epithelial cells and macrophages, *S*. Typhimurium induces mitochondrial fragmentation, altered respiratory capacity, and changes in mtROS production, largely through effector-mediated modulation of host signaling pathways and inflammatory responses (Monack et al., 2004; Lobet et al., 2015). These mitochondrial alterations influence host cell survival and innate immune activation, shaping intracellular bacterial niches and antimicrobial responses. Disruption of mitochondrial bioenergetics during *Salmonella* infection intersects with pathways regulating glucose and lipid metabolism, supporting a potential link between infection-induced inflammation and metabolic dysregulation (Garaude et al., 2016).

*S*. Typhi, which causes a more systemic and often prolonged infection, further illustrates the relevance of sustained mitochondrial stress. Chronic carriage and persistent host-pathogen interactions imply prolonged exposure to inflammatory and metabolic stressors. Although direct experimental evidence linking *S*. Typhi infection to mitochondrial dysfunction remains limited, evidence from chronic inflammation models supports the notion that prolonged mitochondrial perturbation may drive long-lasting metabolic consequences (Dougan and Baker, 2014).

In contrast, *V. parahaemolyticus* is predominantly an extracellular enteric pathogen whose virulence is largely mediated by secreted toxins and type III secretion system effectors acting at the epithelial interface, although intracellular interactions have also been reported (De Souza Santos and Orth, 2014). Similarly to *Salmonella*, *V. parahaemolyticus* induces mitochondrial fragmentation through the activity of the VPA0226 lipase secreted via its type II secretion system and triggers mitochondrial stress responses in epithelial cells through type III secretion system 2-dependent mechanisms (Chimalapati et al., 2020; Plaza et al., 2024). Although direct experimental evidence linking *V. parahaemolyticus* infection to mitochondrial calcium overload, membrane depolarization, or sustained mtROS production remains limited, these downstream mitochondrial phenotypes are biologically plausible outcomes of the types of mitochondrial perturbations induced by bacterial toxins and secretion system effectors.

Together, these pathogens provide a complementary framework to examine how intracellular and predominantly extracellular foodborne bacteria may converge on mitochondria to reshape cellular metabolism and innate immune responses.

## 5 Mitochondrial quality control as a nexus between enteric bacterial infection and long-term metabolic reprogramming: insights from *Salmonella* and *Vibrio*

This stage represents an inflection point in the conceptual model, where mitochondrial quality control mechanisms shape the host’s long-term metabolic trajectory. In bacterial infection, mitochondrial quality control, particularly mitophagy, plays key roles in maintaining cellular homeostasis and regulating inflammatory signaling (Youle and Narendra, 2011; Pickles et al., 2018; Deretic et al., 2013). Mitophagy is predominantly mediated by the PINK1-Parkin pathway, whereby loss of mitochondrial membrane potential stabilizes PINK1 on the outer mitochondrial membrane, promotes Parkin recruitment, and targets dysfunctional mitochondria for autophagic degradation (Youle and Narendra, 2011). Through this mechanism, mitophagy limits excessive mtROS production and restrains mtDAMP release that would otherwise amplify innate immune and inflammatory cascades (West et al., 2011; Nakahira et al., 2015).

During bacterial infection, mitophagy functions as an adaptive host response that clears damaged mitochondria and dampening inflammatory signaling. Experimental evidence from bacterial challenge and lipopolysaccharide-induced inflammation models shows that PINK1/Parkin-dependent mitophagy constrains inflammasome activation and limits tissue injury, whereas impairment of this pathway exacerbates inflammatory damage (Nakahira et al., 2011; Zhong et al., 2016).

Importantly, accumulating evidence indicates that *S.* Typhimurium can actively manipulate host mitophagy and mitochondrial homeostasis during intracellular infection. Recent work shows that host sirtuins, particularly SIRT1 and SIRT3, are engaged during infection to modulate mitochondrial bioenergetics, redox balance, and intravacuolar pH, thereby shaping bacterial survival and replication (Hajra et al., 2025). By altering mitochondrial stress responses and quality control pathways, *Salmonella* modulate host mitophagy-associated mechanisms. In parallel, *S*. Typhimurium has been shown to fine-tune selective autophagy pathways through phosphorylation-dependent activation of TBK1, coordinating mitophagy and xenophagy to restrict excessive bacterial proliferation while preserving host cell viability (Li et al., 2025). This coordinated regulation suggests that mitophagy during *Salmonella* infection is not merely a host-protective response but a dynamically regulated process shaped during infection.

From an immunometabolic perspective, pathogen-driven modulation of mitophagy may have important implications for host metabolic regulation. Experimental studies indicate that dysregulated or maladaptive control of mitochondrial quality may impair stress resolution, leading to sustained mtROS production, altered bioenergetics, and chronic low-grade inflammation, processes that are well stablished contributors to metabolic disorders such as insulin resistance and non-alcoholic fatty liver disease (Hotamisligil, 2017). In the context of bacterial infection, alterations in mitophagy and mitochondrial quality control have been shown to influence inflammatory signaling and cellular homeostasis (Pickles et al., 2018). Together, these observations support a conceptual framework in which *Salmonella*-mediated modulation of mitophagy could contribute to long-lasting immunometabolic reprogramming, without implying direct casual resolution.

To date, there is no direct evidence that *V*. *parahaemolyticus* actively controls host mitophagy pathways. While *V*. *parahaemolyticus* induces pronounced mitochondrial stress through secretion system-dependent mechanisms (Chimalapati et al., 2020; Plaza et al., 2024), whether it modulates mitophagy remains unexplored. Although not yet well explored, whether *Vibrio* infection interferes with mitophagic pathways remains an open mechanistic question that could influence the persistence of mitochondrial dysfunction. Addressing this question could reveal mechanisms by which extracellular enteric pathogens contribute to unresolved mitochondrial stress and post-infectious metabolic vulnerability.

While mitophagy is classically cytoprotective, we propose that maladaptive or unresolved mitophagy during infection may stabilize dysfunctional metabolic states rather than restore homeostasis. Although transient mitophagy is protective, insufficient, excessive, or dysregulated activation can be detrimental, leading to accumulation of dysfunctional mitochondria, sustained mtROS production, and chronic innate immune activation (Pickles et al., 2018). Outside infection context, such mitochondrial dysfunction has been linked to insulin resistance and non-alcoholic fatty liver disease (Hotamisligil, 2017; Sunny et al., 2017). During infection, persistent mitochondrial stress and altered mitophagy may bridge acute pathogen challenge and metabolic dysregulation, as sustained mtROS and mtDAMP signaling interfere with insulin signaling in metabolic tissues. We further propose that mitochondrial stress initiated in epithelial and innate immune cells may propagate systemically via inflammatory mediators, mtDAMPs, and altered metabolite profiles, influencing mitochondrial function and insulin sensitivity in liver, skeletal muscle, and adipose tissue.

Together, these observations position *Salmonella enterica* as a conceptual example of how intracellular pathogens can reshape mitochondrial quality control and influence stress resolution, whereas *V. parahaemolyticus* represents a complementary model in which pronounced mitochondrial stress occurs without reported direct modulation of host mitophagy. This contrast highlights how distinct pathogenic lifestyles may converge on persistent mitochondrial dysfunction through divergent routes, ranging from active reprogramming of quality control pathways to incomplete stress resolution.

Importantly, the central gap addressed in this perspective is not the identification of pathogen-specific mitochondrial mechanisms, but the lack of an integrated framework explaining how infection-induced mitochondrial stress may shape metabolic health. Within this model, secondary questions arise, including pathogen-dependent modulation of mitophagy, determinants of stress resolution versus persistence, and tissue-specific consequences of unresolved mitochondrial damage, defining testable layers for future investigation. Collectively, the evidence supports a model in which mitochondrial stress may act as a modulatory node linking infection-induced inflammation and metabolic regulation, integrating inflammatory, metabolic, and pathogen-derived signals over time and potentially imprinting durable bioenergetic and inflammatory states that shape host metabolic trajectories.

## 6 Conclusions

Chronic low-grade inflammation and metabolic disease are multifactorial processes driven by convergent systemic and tissue-specific stressors, including nutrient excess, adipose dysfunction, gut-derived inflammatory signals, ageing-associated inflammation, and genetic susceptibility (Hotamisligil, 2017). Within this context, mitochondrial stress should not be viewed as a singular causal driver, but rather as a potential amplifier and persistence mechanism through which bacterial infection-induced dysfunction may stabilize inflammatory and metabolic alterations. By framing mitochondria as a modulatory node within infection-induced immunometabolic remodeling, this perspective proposes a conceptual framework linking acute bacterial pathogenesis to long-term metabolic vulnerability and highlights mitochondrial stress resolution as a potential target for mitigating post-infectious metabolic disease.

## Data Availability

The original contributions presented in the study are included in the article/supplementary material. Further inquiries can be directed to the corresponding author.
